# Effect of Oxidation Condition on Growth of N: ZnO Prepared by Oxidizing Sputtering Zn-N Film

**DOI:** 10.1186/s11671-016-1485-7

**Published:** 2016-06-01

**Authors:** Xuesi Qin, Guojian Li, Lin Xiao, Guozhen Chen, Kai Wang, Qiang Wang

**Affiliations:** Key Laboratory of Electromagnetic Processing of Materials (Ministry of Education), Northeastern University, Shenyang, 110819 China

**Keywords:** N-doped ZnO, Film, Oxidation, Growth

## Abstract

Nitrogen-doped zinc oxide (N: ZnO) films have been prepared by oxidizing reactive RF magnetron-sputtering zinc nitride (Zn-N) films. The effect of oxidation temperature and oxidation time on the growth, transmittance, and electrical properties of the film has been explored. The results show that both long oxidation time and high oxidation temperature can obtain the film with a good transmittance (over 80 % for visible and infrared light) and a high carrier concentration. The N: ZnO film exhibits a special growth model with the oxidation time and is first to form a N: ZnO particle on the surface, then to become a N: ZnO layer, and followed by the inside Zn-N segregating to the surface to oxidize N: ZnO. The surface particle oxidized more adequately than the inside. However, the X-ray photoemission spectroscopy results show that the lower N concentration results in the lower N substitution in the O lattice (N_o_). This leads to the formation of n-type N: ZnO and the decrease of carrier concentration. Thus, this method can be used to tune the microstructure, optical transmittance, and electrical properties of the N: ZnO film.

## Background

Zinc oxide (ZnO) has a wide direct bandgap of 3.4 eV at room temperature and a high exciton binding energy of 60 meV [1]. Additionally, it also has features of being low cost, non-toxic, stable, and transparent [2, 3]. The ZnO film has become one of the most important semiconductor materials and exhibits a wide potential application in the fields of light-emitting diodes [4], spintronic device [5], solar cells [6], thermoelectric materials [7, 8], and so on. As a semiconductor material, it is important to form a p-n junction and to increase the concentration and mobility of a carrier. Now, it has been a challenge to build a high-efficiency and stable homo ZnO p-n junction [9] since the carriers are easy to trap at the p-n heterojunction [10, 11]. Although there are reports about the ZnO homo-junction [12–14], a stable and high-efficiency homo-junction is still in the laboratory research stage. The key point is to prepare p-type ZnO and to improve the concentration and mobility of the carrier.

It is generally recognized that the conductivity type of intrinsic ZnO is n-type because there are many donor impurities that exist, such as oxygen vacancy, zinc interstitial, and hydrogen [1, 15]. Furthermore, the concentration and mobility of the carrier in the n-type ZnO is easy to tune by doping the donor impurity (Al, Ga, In, etc.) [16–18]. However, the dopant of acceptor impurity in ZnO is very difficult [19]. Many elements have been selected as the dopant to obtain a more stable p-type ZnO semiconductor, such as N [20, 21], P [22], As [23], Sb [23], Cu [24], Li [25], Na [26], Ag [27], and Au [28]. In addition, a multi-element co-dopant has also been used to improve the acceptor concentration [29, 30] and to increase the whole concentration so as to compensate the intrinsic defect. But this makes the doping theory become complex. At the same time, the doping result is more difficult to control. Because the elements N and O have similar characters of extranuclear electron structure, ionic radius, and shallow acceptor level, N is one of the more effective dopants for obtaining the p-type ZnO [1–3, 21]. However, the problems of low doping concentration and poor stability still remain for the nitrogen-doped zinc oxide (N: ZnO). The effect of the N dopant and microstructure of the ZnO film on the semiconductor properties should be studied further.

In this study, a method has been developed to prepare N: ZnO by thermal oxidation of the reactive radio frequency (RF) magnetron-sputtering zinc nitride (Zn-N) film. Similar oxidation method has been used in our previous studies [31, 32]. That study focuses on the diluted magnetic semiconductor properties of Co: ZnO film by oxidizing a thermal-evaporated Co/Zn bilayer. In this study, the effect oxidation temperature and oxidation time on the crystal structure, surface morphology, chemical state, transmittance, and electrical properties have been studied by using X-ray diffraction (XRD), scanning electron microscopy (SEM), transmission electron microscopy (TEM), X-ray photoemission spectroscopy (XPS), UV–vis spectrophotometry, and the Hall effect measurement. A new growth model of ZnO was found.

## Methods

In this study, a Zn-N precursor film was first prepared by reactive RF magnetron sputtering. A high-purity Zn target (99.99 % purity) was used. High-purity N_2_ (99.99 %) and Ar (99.99 %) were selected as the sputtering gas. The gas partial pressures are 0.3 and 0.5 Pa for N_2_ and Ar, respectively. The base pressure is <2 × 10^−3^ Pa, and the working pressure is 0.8 Pa. The sputtering time is 15 min. The distance between the target and the substrate is 100 mm. RF sputtering power is 100 W. The substrate temperature remains at room temperature. Monocrystal Si(100) and quartz are selected as the substrate. The substrates are cleaned in an ultrasonic device for 15 min and in turn in acetone and alcohol bath and then dried by high-pressure Ar blowing.

The Zn-N films were then oxidized to become N: ZnO films at air atmosphere in a heat treatment furnace. The Zn-N specimens were put into the furnace when the temperature reached the oxidation temperature, and then, the N: ZnO films were obtained by oxidizing for the desired time. In this study, two series of oxidation conditions were considered. (1) The samples were oxidized for 60 min at different oxidation temperatures of 300, 400, 450, and 500 °C. (2) The samples were oxidized at 400 °C for different times at 30, 60, and 120 min.

The crystal structure of the films was examined by XRD (DMAX 2400, Rigaku) with a grazing incidence of 1° in 2*θ* mode with monochromatic Cu Kα1 radiation (*λ* = 0.154056 nm). Surface morphology was examined by SEM (SUPRA 35, Zeiss). The composition and chemical state of the elements were determined by energy-dispersive X-ray spectroscopy (EDX; Inca, Oxford), XPS (ESCALAB 250Xi, Thermo Scientific), and TEM (2100F, JEOL). Optical transmittance was recorded with a UV–vis spectrophotometer (Lambda 750S, PerkinElmer). The electrical property was investigated by the Hall effect measurements (Ecopia HMS-3000, Korea).

## Results and Discussion

**Fig. 1:**
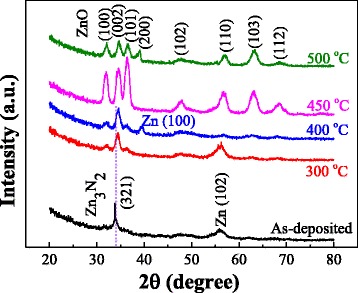
XRD patterns of the as-deposited Zn-N films and N: ZnO films oxidized at different temperatures

In order to confirm the oxidation temperature of ZnO to become transparent for the Zn-N film, the crystal structure and transmittance of the N: ZnO films oxidized at different temperatures for 60 min were first studied. XRD results are shown in Fig. 1. The as-deposited Zn-N film has a (321) peak of Zn_3_N_2_ and a (102) peak of Zn. This means that the as-deposited film is the coexistence of Zn-N and Zn. Furthermore, the position of the (321) peak agrees well with the result of the others [33]. The intensity is different from the result in Ref. [34] and indicates that the as-deposited film of this study has a good crystallinity. After the oxidation, the peak positions are obviously different from those of the as-deposited and exhibit the characters of ZnO. The intensity of the ZnO peak increases with the temperature. The films oxidized at 300 and 400 °C have the peaks of ZnO and Zn. This means that the films are the coexistence of ZnO and Zn and are not oxidized completely. At the same time, the Zn peak changes to Zn (100) at 400 °C from Zn (102) at 300 °C. According to a previous study [35], the preferred orientation of Zn gradually turns to the orientation of the substrate with the increase of oxidation temperature. In this study, the substrate is Si (100); thus, Zn (102) transformed to Zn (002) when the sample was oxidized at 400 °C for 60 min. This means that the oxidation growth is preferred. Then, only peaks of ZnO exist for the films oxidizing at 450 and 500 °C. The Zn-N films were fully oxidized when the oxidation temperature is ≥400 °C. The crystal structure is wurtzite. However, it can be seen that there is no preferred orientation for the N: ZnO film at 500 °C because the temperature is much higher than the melting point of Zn (420 °C).

**Fig. 2:**
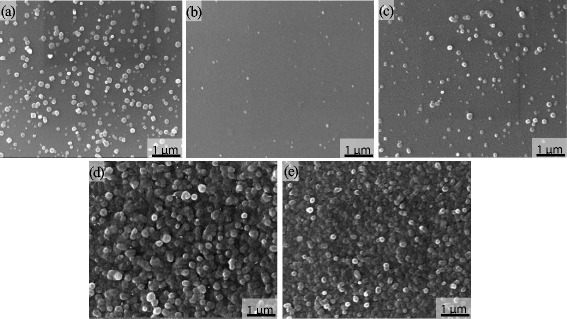
SEM images of the as-deposited film **a** and the films oxidized at different temperatures of **b** 300, **c** 400, **d** 450, and **e** 500 °C for 60 min

SEM was used to analyze the surface morphology of the N: ZnO films, as shown in Fig. 2. Clearly, there are a lot of particles on the surface of the as-deposited Zn-N film. The reason is that some melted droplets of Zn target may be sputtered to the film during the sputtering process because the melting point of Zn is too much low. In addition, there is almost no particle on the surface for the film oxidized at 300 °C. Then, the particle appears again at 400 °C. Moreover, the amount of particles was gradually enhanced with increasing oxidation temperature, and the particles become smaller and aggregate denser. This indicates that the surface morphology can be significantly affected by the oxidation temperature.

**Fig. 3:**
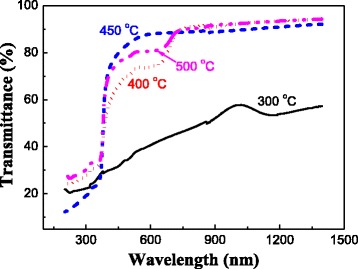
Transmittance of the N: ZnO films oxidized at different temperatures for 60 min

Optical transmittance of the N: ZnO films oxidized at different temperatures were measured to confirm whether the transparency can meet the demand and the films are oxidized fully. The results are shown in Fig. 3. The transmittance in the visible and infrared range of the film oxidized at 300 °C is <60 % because much more Zn exists in the film. The transmittance at the visible range of the film oxidized at other temperatures is > 80 % although their surface morphologies are obviously different. The film oxidized at 450 °C has the best transmittance. This means that the higher amount of particles and denser aggregate did not influence the transmittance. The oxidation degree has a significant effect on the transmittance. Too higher oxidation temperature is not good for the transmittance. Therefore, 400 °C was selected as the temperature to consider the effect of oxidation time on the oxidation growth because the oxidation is not complete within 60 min.

**Fig. 4:**
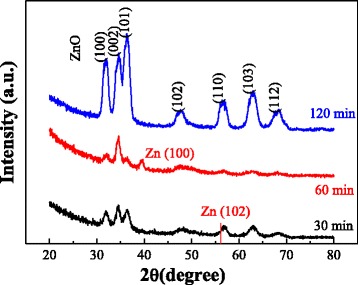
XRD patterns of the N: ZnO films oxidized for different times for 30, 60, and 120 min at 400 °C

Figure 4 shows the XRD patterns of the films oxidized for 30, 60, and 120 min at 400 °C. Clearly, the intensity of ZnO peaks increases with increasing oxidation time. The Zn-N was oxidized completely at 120 min. The position and intensity of the peaks are similar with the film oxidized at 450 °C for 60 min. This indicates that enhancement of both the oxidation temperature and oxidation time can obtain the N: ZnO film with the same crystallinity. Additionally, the XRD peaks of the films oxidized for 30 and 60 min are different. The film oxidized for 30 min has the peaks of ZnO and has no obvious Zn-N and Zn peaks. However, a Zn (100) peak appears for the film oxidized for 60 min. Furthermore, the intensity of the ZnO peaks is also different from that of the peaks oxidized for 30 min. This means that the oxidation growth changes a lot with increasing the oxidation time. More details will be discussed later.

**Fig. 5:**
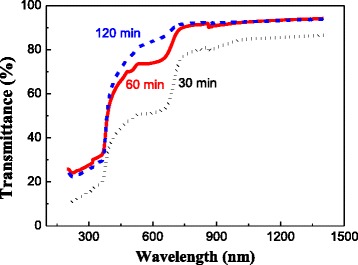
Transmittance of the N: ZnO films oxidized at 400 °C for different times

The optical transmittance of the films oxidized for different times at 400 °C is also shown in Fig. 5. It can be seen that the transmittance increases with increasing the oxidation time. The N: ZnO film oxidized for 120 min has the best transmittance (is >80 % in the visible and infrared range) and is similar with the film oxidized for 60 min at 450 °C. The transmittance is strongly related to the oxidation degree. Both long oxidation time and high temperature can obtain films with a good transmittance. Additionally, the transmittance curves of the films oxidized for 30 and 60 min have a plateau region in the 530–650-nm invisible range. This result is similar with those of the films oxidized at different temperatures. This indicates that the oxidation degree plays a key role in influencing the transmittance. However, this is not the point focused by this study.

**Fig. 6:**
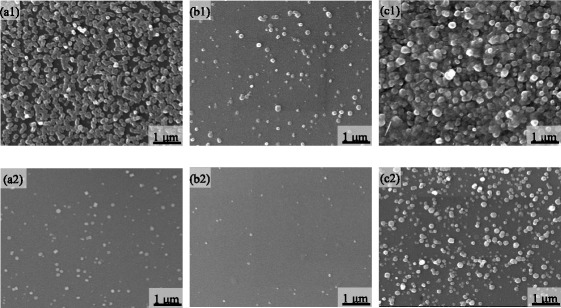
SEM images of the N: ZnO films oxidized for **a** 30, **b** 60, and **c** 120 min at 400 °C (*top row*) and 300 °C (*bottom row*)

SEM was used to test the surface morphologies of the N: ZnO films oxidized at 400 °C for different times, as shown in Fig. 6. It can be seen that the surface is formed by many particles. The amount of the particles on the surface is different for three oxidation times. There are a lot of particles aggregated on the surface of the films oxidized for 30 min. The amount of particles becomes less for 60 min and becomes more for 120 min. Moreover, the surface morphologies of the films oxidized at 300 °C have similar results. The only difference is that the amount of particles is small at a lower oxidation temperature. This indicates that more particles are aggregated on the surface with increasing temperature and time. It is reasonable to conclude that the particle is N: ZnO oxidized by Zn-N. Furthermore, this oxidation growth is significantly different from previous studies [31, 32, 36, 37]. The ZnO grew by forming a dendrite structure in previous studies. This reason may be due to the good crystallinity and existence of a particle on the surface of the as-deposited Zn-N films. Then, why and how to occur this growth are studied.

**Fig. 7:**
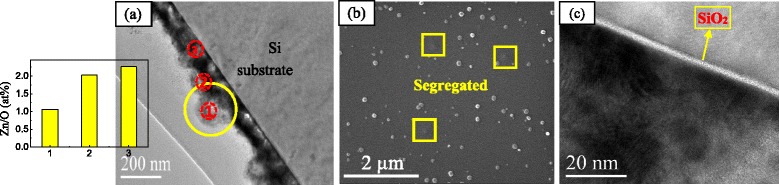
**a** Cross-sectional TEM image and content ratio of Zn/O at different regions for the film oxidized at 400 °C for 60 min. **b** SEM morphology of the film. **c** Cross-sectional HRTEM image for the film oxidized at 400 °C for 60 min

The N: ZnO film oxidized at 400 °C for 60 min is an intermediate process of oxidation growth. TEM and SEM were used to study the cross-sectional structure and composition, as shown in Fig. 7. From the TEM result, the film thickness is about 150 nm. There is an amorphous SiO_2_ layer at the interface between the Si substrate and the film. The thickness and property of this layer is very important for device application of such a heterostructure film [38]. There exist particles on the surface in both TEM and SEM images. The particle size is about 150–200 nm. The content ratio of Zn to O (Zn/O) at the region from the surface to the inside was tested by EDX of TEM. It can be seen that the Zn/O is about 1:1. The Zn ratio increases from the surface to the inside. This means that the particle on the surface oxidized fully. From the morphology in the yellow box of the SEM image, the shape is similar with a mushroom growth from the soil. Thus, it may be the Zn-N particle that segregated from the bottom.

**Fig. 8:**
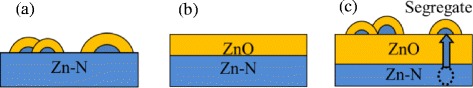
Oxidation growth model of N: ZnO films for different times. **a** Particle on the surface oxidized. **b** Oxidized particles become a N: ZnO layer. **c** Zn-N particles segregate on the N: ZnO surface and oxidized to become a N: ZnO particle

A growth model was proposed based on the above analysis, as shown in Fig. 8. According to a previous study [39], the ZnO growth started from the apex of Zn during the oxidation process. In this study, the particles on the surface of the as-deposited Zn-N film are first oxidized to become N: ZnO particles. This leads to a lot of aggregated particles that exist on the surface of the films oxidized at 400 °C for 30 min. Then, the oxidized N: ZnO particles continue to oxidize until the N: ZnO becomes a continuous layer. This is the reason why the surface becomes smooth for the film oxidized at 60 min. At the third stage, the inside Zn-N particle in the film segregates the surface of N: ZnO because of the existence of voids in the films, as shown by the bright zone in Fig. 7. Similar phenomena were also found in the growth of ZnO nanostructures by wet oxidation method [40]. Then, the segregated Zn-N particles were oxidized to become a N: ZnO particle. Thus, a lot of particles aggregated on the surface of the film at 120 min. This leads to the adequate oxidation of the particles on the surface.

**Fig. 9:**
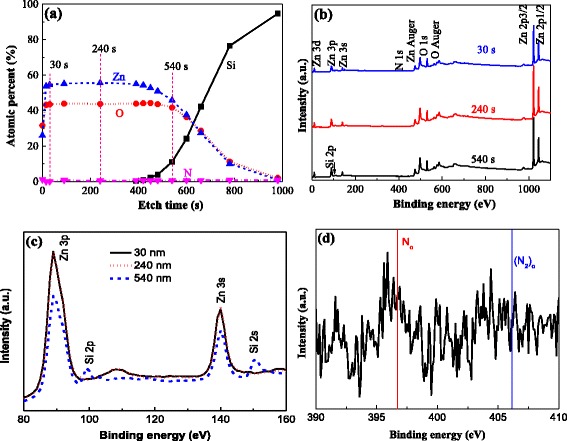
XPS spectra of N: ZnO film oxidized at 400 °C for 60 min. **a** Depth profile. **b** Survey scan spectra at 30, 240, and 540 s. High-resolution XPS of **c** Si and **d** N at 240 s

XPS was used to examine the composition in depth and the chemical states of the elements. The results are shown in Fig. 9. From the depth profile, it can be seen that Zn, O, and N distribute uniformly in the depth. The Zn content is higher than that of O because the film was not oxidized fully. The N content is very low and about 0.65 %. Additionally, the case of a higher Zn ratio versus O on the surface, as shown in the TEM result, has not appeared in the XPS. This may be because the point to measure the content is not at the particle position during the sputtering measure process. Then, the XPS survey scan spectra with different etching times of 30, 240, and 540 s were used to define the chemical states of the elements, as show in Fig. 9b. Clearly, three peaks have no significant difference. All peaks can be ascribed to the elements Zn, O, and N. This means that there is no impurity in the film. However, there is an obvious difference for the high-resolution XPS of Si, as show in Fig. 9c. The Si 2p and 2s peaks appear because the Si of the substrate diffuses at the interface of the N: ZnO and Si substrates. This leads to the increase of Si and decrease of Zn and O in the etching time of 400–700 s in Fig. 9a. For the high-resolution XPS of N at 240 s, there are two peaks of N 1s that appear at 396.7 and 405.1 eV [41]. They depict, respectively, the concentration of the N_o_ acceptor and (N_2_)_o_ double donor. The peak intensity is weak because the content of N is low. Additionally, when the intensity of N_o_ is much stronger than that of (N_2_)_o_, the N: ZnO film can become n-type.

Then, the Hall effect measurement was used to test the electrical properties of the N: ZnO films oxidized at 400 °C for different times, as shown in Table 1. The Hall coefficients of all the films are negative. This means that all the films are n-type. The p-type N: ZnO was not formed because the N content is too low, as the above analysis of the N XPS result. Furthermore, the carrier concentration of the film oxidized for 60 min is 2 orders of magnitude lower than the others. The reason may be as follows. For the film oxidized for 30 min, more Zn can provide more carriers. For the film oxidized for 120 min, more O substitution by N can provide more carriers. In addition, the carrier mobility of the film oxidized for 60 min is higher than that of the others. This is because the smooth N: ZnO layer was formed at this stage. This indicates that the aggregation of the N: ZnO particles can deteriorate the carrier mobility because of the scatter of carrier by particle boundaries. This result illustrates that the oxidation method can affect the growth and microstructure. The correlation of structure and electrical properties demonstrates that the oxidation growth gives an effective method to obtain the needed structure and properties of the N: ZnO film.

**Table 1:** Electrical properties of N: ZnO film oxidized at 400 °C for different times

Samples (min)	Resistivity (Ω cm)	Carrier mobility (cm^2^/V S)	Concentration (cm^−3^)	Hall coefficient (cm^3^/C)
30	7.15	0.92	9.44 × 10^17^	−6.61
60	449.7	2.03	6.82 × 10^15^	−916.1
120	28.01	1.36	1.64 × 10^17^	−38.19

However, this kind of growth proposes a new method to control the N: ZnO structure. Furthermore, there is a big difference for the carrier concentration and mobility at different oxidation stages. These provide possibility to prepare p-type ZnO and to improve the concentration and mobility of the carrier. In our study, there are several reasons that lead to the n-type formation and the low carrier concentration and mobility. (1) The substrate temperature maintains at room temperature the deposition of the as-deposited Zn-N film in this study. There are a lot of donor impurities (oxygen vacancy, interstitial zinc, etc.) [42], which lead to a strong self-compensation effect after oxidation. (2) N content is too low. The lower N concentration results in the lower N substitution in the O lattice (N_o_). This leads to the formation of n-type N: ZnO and the decrease of carrier concentration. In addition, this leads to the electrical properties of the films which are similar with those of the pure ZnO thin film (n-type) and also the lower carrier concentration [43]. Furthermore, the insulating quartz substrate is nonconductive to generate p-type semiconductors [44].

## Conclusions

The N: ZnO film was fabricated by the thermal oxidation of the reactive RF magnetron-sputtering Zn-N film. Oxidation temperature and oxidation time have a significant effect on the crystal structure, surface morphology, and chemical state. The correlation of the optical transmittance and electrical properties with the structure was explored. The results show that the control of oxidation condition of N: ZnO film has made the film exhibit a special growth model. The 400 °C temperature and 120 min time is the best oxidation condition for obtaining a high-quality N: ZnO film. The film can be oxidized completely at this condition. Meanwhile, its transmittance is over 85 % for the visible and infrared light and has a higher carrier concentration. The lower N concentration results in the lower N_o_ substitution in the O lattice. This leads to the formation of n-type N: ZnO and the decrease of carrier concentration. This oxidation growth gives an effective method to obtain the needed structure and properties of the N: ZnO film.
